# The risk assessment of sleep duration on geriatric sarcopenia and its regulatory role in the effect of BMI index on geriatric sarcopenia based on the CHARLS database

**DOI:** 10.1371/journal.pone.0345257

**Published:** 2026-03-20

**Authors:** Yan Chen, Xue-Ke Shi, Nan-Nan Liu, Zhihai Feng

**Affiliations:** 1 Rehabilitation Center, The First Affiliated Hospital of Henan University of Chinese Medicine, Zhengzhou, China; 2 Department of endocrinology, The First Affiliated Hospital of Henan University of Chinese Medicine, Zhengzhou, China; Nanjing Medical University, CHINA

## Abstract

**Background:**

The effect of sleep duration on geriatric sarcopenia remains unclear, and evidence of the combined impact of it and BMI on geriatric sarcopenia is scarce.

**Aims:**

To conduct the risk assessment of sleep duration on geriatric sarcopenia and explore its moderating role in the effect of BMI on geriatric sarcopenia based on the CHARLS database.

**Methods:**

Data were extracted from the China Health and Retirement Longitudinal Study (CHARLS) 2011, 2013, and 2015 waves respectively. Logistic regression and Cox regression analyses were conducted to assess the risk of sleep duration for geriatric sarcopenia, and the regulatory effect of sleep duration on the association between BMI and sarcopenia. Subgroup analysis was conducted to enhance the stability of the results.

**Results:**

The four abnormal types of sleep duration were significantly associated with sarcopenia. A significant association between obesity and decreased risk of sarcopenia was observed in sufficient sleep and sleep deprivation groups after the full adjustment of covariates (Adjusted HR = 0.25, 95%CI: 0.10–0.64, P < 0.001; Adjusted HR = 0.13, 95%CI: 0.03–0.53, P = 0.004; Adjusted HR = 0.26, 95%CI: 0.10–0.66, P = 0.005), and relationships of underweight and overweight with sarcopenia were observed in sufficient sleep, sleep deprivation, and mild oversleeping groups. In the group of oversleeping, the associations were not significant.

**Conclusion:**

Abnormal sleep durations including sleep deprivation and oversleeping are linked to a heightened risk of sarcopenia among older adults. Underweight is associated with the increased sarcopenia risk, and overweight is correlated with the decreased risk of sarcopenia, especially in females with abnormal sleep duration except for severe oversleeping, and obesity is associated with the decreased risk of sarcopenia, especially in adults aged over 65 with sleep deprivation.

## Introduction

In recent years, population aging has become a significant global concern. Maintaining muscle mass, strength, and function during aging is paramount for maintaining health, independence, and quality of life. Extensive research has demonstrated that after the age of 50, adults experience an annual decline in muscle mass and strength of approximately 1%, with this rate accelerating to about 3% beyond age 60 [1]. This ageassociated clinical condition, now classified as a disease in the International Classification of Diseases (ICD10 CM code M62.84), is characterized by the progressive and widespread loss of skeletal muscle mass, diminished muscle strength, and/or impaired physical function, and is referred to as sarcopenia [2]. Global estimates suggest that approximately 10% to 16% of the general elderly population is affected by sarcopenia, with even higher prevalence rates observed among patients [3]. As global life expectancy rises and fertility rates decline, the prevalence of sarcopenia is expected to increase annually. This pathology escalates the risk of falls, fractures, functional decline, comorbidities, and even mortality, significantly undermining the quality of life for older adults. It also diminishes well-being and independence, while imposing a significant economic burden on families and society [4]. Therefore, it is imperative to identify the risk factors for sarcopenia prevention.

Among the various factors influencing sarcopenia, sleep duration has increasingly drawn attention. Although multiple studies have investigated the relationship between sleep duration and sarcopenia, the evidence remains inconsistent. For instance, Chen et al. reported that sleep deprivation significantly increased the risk of sarcopenia in middle-aged and elderly Chinese populations [5]. This finding contrasts with a study from Japan, which concluded that oversleeping is strongly associated with a higher risk of sarcopenia in older adults [6]. In contrast, Li et al. highlighted that both sleep deprivation and oversleeping were positively associated with the risk of sarcopenia, with this relationship being particularly evident in the elderly population [7]. Additionally, Pourmotabbed et al. further predicted that eight hours of sleep per day is considered the optimal duration for minimizing the risk of sarcopenia. Notably, sex appears to play a role in this relationship [8]. Lee et al. further demonstrated that oversleeping was linked to sarcopenia in men, while no significant effect of sleep duration on sarcopenia was observed in women [9]. Thus, a deeper understanding of the interaction between sleep duration and sarcopenia is crucial for developing targeted strategies and interventions.

BMI is frequently regarded as closely linked to muscle mass and strength [10]. Most opinions suggest that higher BMI is inversely associated with the risk of sarcopenia, aligning with the obesity paradox, which posits that being overweight or obese may offer certain health benefits for the elderly population [11–13]. However, Hashim et al. analysis showed that BMI ≥ 30 kg/m2 was associated with a high risk of sarcopenia [14]. Concerning the relationship between sleep duration and BMI, previous research has identified sleep deprivation as an independent predictor of high BMI [15,16]. An analysis of data from the National Health and Nutrition Examination Survey (NHANES) revealed that the relationship between sleep duration and BMI varies by age group: it is negatively linear in young adults, U-shaped in middle-aged adults, and weak in older adults [17]. Moreover, a study by Shochat et al. found an inverted J-shaped relationship between BMI and sleep duration in older women, where those with both low and high BMI experienced shorter sleep durations [18]. However, the moderating role of sleep duration on the relationship between BMI and sarcopenia remains unclear. Therefore, we investigated the impact of sleep duration and BMI on sarcopenia in a cohort of elderly Chinese adults respectively, and explored the potential moderating effect of sleep duration on the relationship between BMI and sarcopenia risk.

## Methods

### Study population

The CHARLS is a nationally representative longitudinal study designed to collect comprehensive data on the aging population in China. CHARLS aims to provide insights into the health, economic, and social circumstances of individuals aged 45 and older in China. It serves as a valuable resource for researchers studying aging, health care, economics, and social issues. The first wave of data collection began in 2011, and subsequent waves have been conducted approximately every two years. The study uses a structured questionnaire that covers various domains, including Health status and medical history, Functional ability, Cognitive function, Economic status (income, expenditure, assets), Social engagement and support, health care access and utilization. CHARLS initially surveyed over 17,000 individuals across 150 counties in 28 provinces, making it one of the largest studies of its kind in China. The CHARLS data is publicly available for researchers, enabling them to conduct analyses and contribute to the understanding of aging issues in China. The dataset is accessible through the CHARLS official website (http://charls.pku.edu.cn/index.htm).

**Fig 1:**
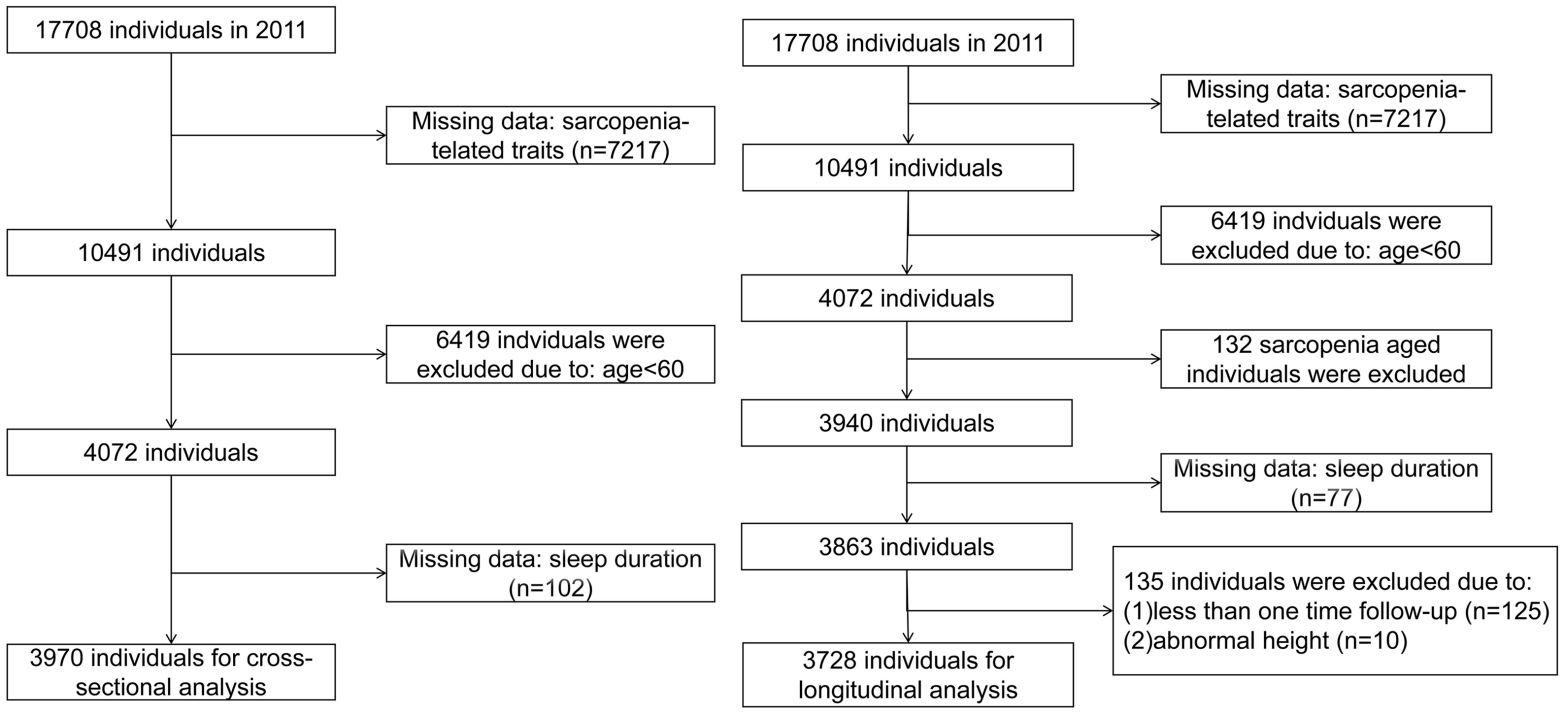
Flowchart of the sample selection process.

This study utilized 2011, 2013, and 2015 waves to conduct longitudinal cohort analysis, the exclusion criteria are as follows: [1] individuals aged < 60 years old; [2] missing sarcopenia-related traits index; [3] missing sleep duration data; [4] individuals with sarcopenia in 2011; [5] missing follow-up for two times. And we conducted cross-sectional logistic regression analysis using data from CHARLS 2011, and the exclusion criteria are as follows: [1] individuals aged < 60 years old; [2] missing sarcopenia-related traits index; [3] missing sleep duration data. The selection details are shown in Fig 1.

In this study, abnormal values like height = 0.7m were removed to ensure the accuracy of the analysis results. Ultimately, a total of 3728 individuals were included in the baseline analysis.

### Ethics approval

The CHARLS was ethically approved by the institutional review board at Peking University (Nu: IRB00001052–11015).

### Assessment of sarcopenia

The diagnosis of sarcopenia refers to the criteria proposed by the 2019 Asian Working Group for Sarcopenia (AWGS). Sarcopenia is defined through muscle strength, muscle mass, and physical function. Muscle strength is reflected through grip strength assessed by a dynamometer. Muscle strength decline is defined as grip strength < 28 kg for men and < 18 kg for women. For muscle mass, the CHARLS study did not measure the muscle mass of respondents. This study estimated appendicular skeletal muscle mass (ASM) based on the equation expressed as ASM = 0.193 * body weight (kg) + 0.107 * height (cm) − 4.157 * gender (male = 1, female = 2) − 0.037 * age − 2.631 [19]. Muscle mass is also assessed using height-adjusted ASM (ASM/height²), with values < 7.0 kg/m² for men and < 5.4 kg/m² for women indicating a decline in muscle mass. Physical function is evaluated using a 2.5-meter walking test: participants are instructed to walk a straight 2.5-meter path twice (round trip) at their usual pace, and the average speed is calculated. A walking speed < 1 m/s indicates a decline in physical function. Sarcopenia is defined as a decline in muscle mass combined with physical function or a decline in muscle mass combined with muscle strength.

### Sleep duration

Data on sleep duration were collected by self-report through the question: “How many hours did you fall asleep each night on average in the past month?” in the questionnaire. Based on the sleep duration recommendations from the National Sleep Foundation (NSF), sleep durations can be categorized into five levels according to baseline age and sleep time. The details of the groups are shown in Table 1.

**Table 1:** Groups for sleep duration.

	Sleep duration (h)				
age	Severe sleep deprivation	Mild sleep deprivation	Sufficient sleep	Mild oversleeping	Severe oversleeping
60-65	<6	6-7	7-9	9-10	>10
>65	<5	5-7	7-8	8-9	>9

### BMI index

BMI was measured by body weight and height, with the equation: BMI = body weight (Kg)/height^2^ (m^2^), and it was treated as a categorical variable for the analysis.

### Covariates

We selected demographic characteristics including age, sex, marital status (married and unmarried), education level (no formal education illiterate, elementary school, middle school, and college or above), residence status (rural and urban), health-related factors including body mass index (BMI) calculated by body weight (Kg)/height (m^2^), waist circumference, and physical activity, and laboratory indexes including glucose, creatinine, triglycerides (TG), high-density lipoprotein cholesterol (HDL-c), low-density lipoprotein cholesterol (LDL-c), and C-response protein (CRP) as covariates of this study.

### Statistical analysis

Data cleansing and analysis were conducted using Stata 17.0 and R 4.4.1. Baseline data extracted in the 2011 wave were analyzed to assess the baseline characteristics of the participants. All variables were grouped by five sleep duration types (severe sleep deprivation, mild sleep deprivation, sufficient sleep, mild oversleeping, and severe oversleeping), continuous variables were presented by mean and standard deviation (SD) or median (Interquartile range, IQR), and categorical variables were presented by frequencies (percentages). Continuous variables among groups were compared using one-way analysis of variance (ANOVA) or Kruskal–Wallis test, and we conducted Bonferroni correction for multiple comparisons. The chi-square test was applied to compare the difference in categorical variables among groups. Logistic and Cox regression models were applied to estimate the relationship of sleep duration and BMI with sarcopenia. Model 1 was not adjusted; Model 2 was adjusted for age, sex, marital status, education level, residence status, BMI, waist circumference and physical activity; Model 3 was further adjusted for glucose, creatinine, HDL-c, LDL-c, TG, and CRP based on Model 2. A stratified analysis was conducted to evaluate the moderating effect of sleep duration on the association of BMI with sarcopenia. Odds ratios (ORs), Hazard Ratios (HRs), and their corresponding 95% confidence intervals (CIs) were calculated from the regression Models. P-value < 0.05 was considered to have a statistical difference.

## Results

### Baseline characteristics of participants in 2011

A total of 3728 individuals participated in this study, and males accounted for 47.72%. All participants were divided into five groups based on their sleep duration: sufficient sleep, severe sleep deprivation, mild sleep deprivation, mild oversleeping, and severe oversleeping. For missing values, we utilized multiple imputation methods to complete the data. The details of baseline characteristics are presented in Table 2, and the details of missing data are shown in Supplementary Materials. The partcipants characteristics for cross-sectional analysis are presented in Supplementary Materials. P-values were settled with the Bonferroni method. Significant difference of sex, age, residence status, marital status, education level, serum creatinine, glucose, and HDL-c was observed among groups of sleep duration.

**Table 2:** Baseline characteristics of participants in 2011.

Characteristics	Total	Sleep time groups					Bonferroni’s P
		Sufficient sleep	Severe sleep deprivation	Mild sleep deprivation	Mild oversleeping	Severe oversleeping	
Number of people	3728	962	1014	1046	607	99	/
Sex, n (%)							<0.001
Female	1950	500 (49.60)	631 (60.56)	498 (48.92)	202 (46.44)	119 (52.89)	
Male	1778	479 (49.79)	397 (39.15)	534 (51.05)	319 (52.55)	49 (49.49)	
Age (year), mean ± SD	3728	64.74 ± 4.64	65.97 ± 5.56	67.27 ± 5.56	68.92 ± 5.34	70.86 ± 5.36	<0.001
BMI (Kg/m^2^), mean ± SD	3728	23.44 ± 3.82	22.65 ± 5.56	23.08 ± 3.93	23.13 ± 4.02	22.49 ± 3.72	0.137
Waist circumference(cm), mean ± SD	3728	85.27 ± 11.97	83.06 ± 12.09	84.47 ± 12.78	85.48 ± 11.71	83.90 ± 11.94	0.690
Residence, n (%) < 0.001							
rural	2526	641 (66.63)	715 (70.51)	661 (63.19)	428 (70.51)	81 (81.82)	
urban	1202	321 (33.37)	299 (29.49)	385 (36.81)	179 (29.49)	18 (18.18)	
Education level, n (%) < 0.001							
No formal education illiterate	2118	511 (53.17)	658 (64.89)	528 (50.48)	353 (58.15)	68 (68.69)	
Elementary school	960	262 (27.26)	247 (24.36)	265 (25.36)	162 (26.69)	24 (24.24)	
Middle school	455	124 (12.90)	86 (8.48)	172 (16.46)	67 (11.04)	6 (6.06)	
College or above	195	64 (6.66)	24 (2.37)	81 (7.75)	25 (4.12)	1 (1.01)	
Marital status, n (%)							<0.001
Married	3077	877 (87.00)	842 (80.81)	843 (82.81)	348 (80.00)	167 (74.22)	
Unmarried	651	131 (13.00)	200 (19.19)	175 (17.19)	87 (20.00)	58 (25.78)	
Glucose (mg/dl), mean ± SD	3728	112.09 ± 32.86	109.64 ± 28.89	111.36 ± 32.15	110.97 ± 30.38	105.96 ± 17.87	0.035
Creatinine (mg/dl), mean ± SD	3728	0.81 ± 0.18	0.78 ± 0.16	0.80 ± 0.19	0.81 ± 0.17	0.82 ± 0.19	0.003
TG (mg/dl), mean ± SD	3728	130.19 ± 107.56	125.20 ± 77.70	124.00 ± 73.98	128.04 ± 88.33	130.85 ± 83.88	0.563
Hdl Cholesterol (mg/dl), mean ± SD	3728	51.65 ± 14.65	53.44 ± 14.54	52.35 ± 14.17	51.12 ± 13.15	53.67 ± 16.19	0.009
Ldl Cholesterol (mg/dl), mean ± SD	3728	119.22 ± 31.79	118.49 ± 31.71	119.51 ± 32.65	120.35 ± 32.97	115.50 ± 33.23	0.777
CRP (mg/L), median (IQR)	3728	1.55 (0.70, 2.62)	1.51 (0.67, 2.62)	1.52 (0.72, 2.62)	1.81 (0.74, 2.62)	1.70 (0.74, 2.62)	0.544
Physical activity, n (%)							0.537
No	677 (18.16)	187 (18.55)	184 (17.66)	181 (17.78)	86 (19.77)	39 (17.33)	
Light	959 (25.72)	252 (25.00)	262 (25.14)	263 (25.83)	127 (29.20)	55 (24.44)	
Moderate	1041 (27.92)	277 (27.48)	317 (30.42)	276 (27.11)	103 (23.68)	68 (30.22)	
Vigorous	1051 (28.19)	292 (28.97)	279 (26.78)	298 (29.27)	119 (27.36)	63 (28.00)	

SD, standard deviation; n (%), count (percentage); IQR, Interquartile range; BMI, body mass index; TG, triglycerides; HDL-c, high-density lipoprotein cholesterol; LDL-c, low-density lipoprotein cholesterol; CRP, C-response protein.

### Logistic regression of association between sleep duration and sarcopenia

To further investigate the association of sleep duration with sarcopenia, we conducted logistic regression analysis by using three models, with sarcopenia treated as a binary variable (“sarcopenia” = 1, “without sarcopenia” = 0). The results shown in Table 3 revealed that severe sleep deprivation, mild sleep deprivation, mild oversleeping, and severe oversleeping were all associated with sarcopenia compared to sufficient sleep in the three models.

**Table 3:** Logistic regression results of relationship between sleep duration and sarcopenia.

Characteristics	Model 1			Model 2			Model 3		
	OR	95%CI	P	OR	95%CI	P	OR	95%CI	P
Sleep duration									
Sufficient sleep	ref			ref			ref		
Severe sleep deprivation	2.06	1.71, 2.49	<0.001	1.74	1.39, 2.18	<0.001	1.97	1.31, 2.95	0.001
Mild sleep deprivation	1.51	1.25, 1.83	<0.001	1.55	1.23, 1.95	<0.001	1.70	1.13, 2.57	0.010
Mild oversleeping	1.53	1.19, 1.95	<0.001	1.68	1.25, 2.25	<0.001	2.58	1.53, 4.38	<0.001
Severe oversleeping	1.93	1.42, 2.62	<0.001	2.00	1.39, 2.88	<0.001	2.89	1.52, 5.51	0.001

OR, odds ratio; CI, Confidence Interval

Model 1 was not adjusted;

Model 2 was adjusted for age, sex, marital status, education level, residence status, BMI, waist circumference and physical activity;

Model 3 was further adjusted for glucose, creatinine, HDL-c, LDL-c, TG, and CRP based on Model 2.

### Cox regression analysis of association between sleep duration and sarcopenia risk

We explored the relationship of sleep duration with the risk of sarcopenia, with the results shown in Table 4. The findings indicated that severe sleep deprivation, mild sleep deprivation, mild oversleeping, and severe oversleeping were associated with sarcopenia risk compared to sufficient sleep in the three models. To enhance the stability of the results, subgroup analysis was performed to explore the difference of the relationship between sleep duration and sarcopenia in different sex. As shown in Supplementary Materials, no significant interaction between sleep duration and sex was observed, although the significant association of sleep duration with sarcopenia was only observed in females after the full adjustment of covariates.

**Table 4:** Cox regression results of relationship between sleep duration and sarcopenia.

Characteristics	Model 1			Model 2			Model 3		
	HR	95%CI	P	HR	95%CI	P	HR	95%CI	P
Sleep duration									
Sufficient sleep	ref			ref			ref		
Severe sleep deprivation	1.35	1.09, 1.66	0.005	1.31	1.06, 1.62	0.012	1.29	1.04, 1.59	0.020
Mild sleep deprivation	1.33	1.08, 1.65	0.008	1.34	1.08, 1.65	0.007	1.32	1.07, 1.63	0.011
Mild oversleeping	1.55	1.20, 2.00	<0.001	1.41	1.09, 1.82	0.009	1.40	1.08, 1.81	0.010
Severe oversleeping	1.70	1.25, 2.31	<0.001	1.49	1.09, 2.03	0.012	1.49	1.10, 2.03	0.011

HR, Hazard ratio; CI, Confidence Interval

Model 1 was not adjusted;

Model 2 was adjusted for age, sex, marital status, education level, residence status, BMI, waist circumference and physical activity;

Model 3 was further adjusted for glucose, creatinine, HDL-c, LDL-c, TG, and CRP based on Model 2.

### Association between BMI and risk for sarcopenia, stratified by sleep duration

We hypothesized that sleep duration moderates the association of BMI with the risk of sarcopenia. To test this, we performed an analysis stratified by BMI categories (underweight: < 18.5, normal weight: 18.5−24, overweight and obesity: 24–27.9, obesity: ≥ 28). As shown in Table 5, overweight and obesity were associated with a decreased risk of sarcopenia across all individuals in the three models (Adjusted HR = 0.49, 95%CI:0.39–0.63, P < 0.001; Adjusted HR = 0.31, 95%CI: 0.19–0.51, P < 0.001), and underweight was associated with a increased risk of sarcopenia (Adjusted HR = 1.67, 95%CI: 1.36–2.06, P < 0.001). When the analysis was stratified by sleep duration, a significant association between obesity and decreased risk of sarcopenia was observed in sufficient sleep and sleep deprivation groups after the full adjustment of covariates (Adjusted HR = 0.25, 95%CI: 0.10–0.64, P < 0.001; Adjusted HR = 0.13, 95%CI: 0.03–0.53, P = 0.004; Adjusted HR = 0.26, 95%CI: 0.10–0.66, P = 0.005), and relationships of underweight and overweight with sarcopenia were observed in sufficient sleep, sleep deprivation, and mild oversleeping groups. In the group of oversleeping, the associations were not significant.

**Table 5:** Association between BMI and risk of sarcopenia, stratified by sleep duration.

Characteristics	Model 1			Model 2			Model 3			No. of cases/total
	HR	95%CI	P	HR	95%CI	P	HR	95%CI	P	
All participants										
Normal BMI	ref			ref			ref			468/2022
Underweight	1.99	1.64, 2.41	<0.001	1.70	1.38, 2.09	<0.001	1.67	1.36, 2.06	<0.001	132/339
Overweight	0.36	0.29, 0.45	<0.001	0.48	0.38, 0.61	<0.001	0.49	0.39, 0.63	<0.001	95/1014
Obesity	0.20	0.13, 0.32	<0.001	0.30	0.19, 0.49	<0.001	0.31	0.19, 0.51	<0.001	19/353
Sufficient sleep										
Normal BMI	ref			ref			ref			85/498
Underweight	2.25	1.45, 3.50	<0.001	2.28	1.37, 3.79	0.002	2.33	1.40, 3.88	0.001	23/65
Overweight	0.45	0.29, 0.70	<0.001	0.51	0.31, 0.84	0.008	0.50	0.31, 0.83	0.007	27/292
Obesity	0.27	0.12, 0.61	0.002	0.26	0.10, 0.66	0.005	0.25	0.10, 0.64	0.004	6/107
Severe sleep deprivation										
Normal BMI	ref			ref			ref			135/577
Underweight	2.08	1.48, 2.92	<0.001	1.60	1.11, 2.29	0.012	1.55	1.07, 2.24	0.020	43/111
Overweight	0.32	0.20, 0.50	<0.001	0.37	0.23, 0.59	<0.001	0.39	0.24, 0.63	<0.001	23/245
Obesity	0.09	0.02, 0.37	<0.001	0.12	0.03, 0.51	0.004	0.13	0.03, 0.53	0.004	1/81
Mild sleep deprivation										
Normal BMI	ref			ref			ref			138/567
Underweight	1.91	1.31, 2.79	<0.001	1.86	1.23, 2.79	0.003	1.77	1.17, 2.70	0.008	35/97
Overweight	0.37	0.25, 0.56	<0.001	0.55	0.35, 0.85	0.007	0.55	0.35, 0.85	0.008	28/281
Obesity	0.17	0.07, 0.40	<0.001	0.26	0.10, 0.66	0.005	0.26	0.10, 0.66	0.005	6/101
Mild oversleeping										
Normal BMI	ref			ref			ref			88/324
Underweight	2.36	1.41, 3.95	0.001	2.37	1.35, 4.22	0.004	2.35	1.31, 4.21	0.004	26/53
Overweight	0.27	0.14, 0.50	<0.001	0.37	0.19, 0.75	0.005	0.37	0.18, 0.76	0.007	15/173
Obesity	0.20	0.06, 0.64	0.007	0.39	0.11, 1.33	0.132	0.36	0.10, 1.28	0.113	3/57
Severe oversleeping										
Normal BMI	ref			ref			ref			22/56
Underweight	1.63	0.81, 3.29	0.172	1.64	0.79, 3.41	0.188	1.57	0.73, 3.34	0.247	5/13
Overweight	0.30	0.13, 0.72	0.007	0.53	0.21, 1.32	0.171	0.52	0.20, 1.30	0.161	2/23
Obesity	0.66	0.20, 2.15	0.488	1.42	0.42, 4.84	0.571	1.41	0.39, 5.11	0.597	3/7

HR, Hazard Ratio; CI, Confidence Interval.

Model 1 was not adjusted;

Model 2 was adjusted for age, sex, marital status, education level, residence status, waist circumference and physical activity;

Model 3 was adjusted for age, sex, marital status, education level, residence status, waist circumference, physical activity, glucose, creatinine, HDL-c, LDL-c, TG, and CRP.

We then conducted subgroup analyses based on sex and age, as shown in Supplementary Materials. The negative relationship between overweight and sarcopenia risk was only observed in females, and the positive association between underweight and sarcopenia was only observed in females with abnormal sleep duration except for severe oversleeping, and males with sufficient sleep. Additionally, overweight was associated with the decreased risk of sarcopenia only in participants aged over 65 with sleep deprivation. For both subgroup analyses of sex and age, association of BMI and sarcopenia was not significant.

## Discussion

In this study of adults aged 60 and older, abnormal sleep duration, including severe and mild sleep deprivation as well as mild and severe oversleeping, were significantly associated with sarcopenia compared to the sufficient sleep group. Although findings on the relationship between sleep duration and sarcopenia has been mixed, our analysis aligns with a meta-analysis indicating that both insufficient and excessive sleep are closely linked to sarcopenia progression [8]. However, studies conducted in Korea and Japan concluded that oversleeping increases the prevalence of sarcopenia, while sleep deprivation shows no association with its progression [6,20]. This finding contrasts with the conclusions of Chen et al., who reported that the prevalence of sarcopenia decreases with longer sleep durations [5]. The conflicting results may stem from the age of the subjects included in the study, the sample size, the definition of sleep duration categories, and various confounding factors. Therefore, larger prospective cohort studies are needed in the future to clarify the specific relationship between sleep duration and sarcopenia.

A cohort study involving 1,902 participants found that objectively measured short sleep duration (<6.2 hours) and long sleep duration (>8.5 hours) were both significantly associated with sarcopenia (OR = 1.74, 95% CI = 1.07–2.82; OR = 6.66, 95% CI = 3.45–12.87) [21]. Although that study defined sleep duration differently by using objective measurements rather than self-reported questionnaires, its results still suggest that both sleep deprivation and oversleeping contribute to sarcopenia. Another prospective study similarly indicates that both insufficient and excessive sleep increase the risk of low muscle strength, reduced muscle mass, and sarcopenia [22].

BMI is an important risk factor for sarcopenia. In a cross-sectional study [23], the average BMI of participants without sarcopenia is 25.24 ± 3.42 kg/m², while the average BMI of participants with sarcopenia is 20.58 ± 2.30 kg/m², suggesting that a higher BMI may have a protective effect against the risk of sarcopenia. Although the influence of sleep duration on sarcopenia has been studied, it remains unclear whether sleep duration modulates the relationship between BMI and sarcopenia. Therefore, this study analyzed the role of sleep duration in moderating the effect of BMI on the risk of sarcopenia.

The increased risk of sarcopenia associated with abnormal sleep durations stems from multifaceted disruptions to neuromuscular homeostasis, with mechanisms operating at molecular, cellular, and systemic levels. Sleep deprivation fundamentally dysregulates the hypothalamic-pituitary-adrenal (HPA) axis, causing sustained elevation of cortisol that directly activates muscle proteolysis through ubiquitin-proteasome and autophagy-lysosome pathways, which occurs via upregulation of the transcription factors Forkhead Box O1 (FoxO1) and Forkhead Box O3 (FoxO3), which stimulate E3 ubiquitin ligases, while concurrently suppressing mechanistic target of rapamycin complex 1 (mTORC1)-mediated protein synthesis [24–26]. Simultaneously, sleep fragmentation impairs slow-wave sleep, blunting growth hormone (GH) pulsatility and reducing hepatic and intramuscular Insulin-like growth factor 1 (IGF-1) production, which are critical anabolic signals for satellite cell activation and myonuclear accretion [27,28]. Additionally, sleep loss induces insulin resistance through c-Jun N-terminal kinase 1 (JNK-1)-mediated serine phosphorylation of Insulin Receptor Substrate 1 (IRS-1), impairing Glucose transporter type 4 (GLUT4) translocation and glucose uptake in skeletal muscle [29]. Sleep deprivation also elevates pro-inflammatory cytokines via nuclear factor kappa-B (NF-κB) activation in adipose tissue, creating systemic inflammation that directly triggers muscle wasting through signal transduction and activation of transcription 3 (STAT3)-dependent myostatin upregulation [30].

Oversleeping, particularly severe forms, reflects underlying pathologies that exacerbate sarcopenia through distinct mechanisms. Extended supine positioning reduces mechanical loading, downregulating mechanotransduction pathways essential for maintaining muscle architecture [31]. Prolonged sleep also correlates with attenuated diurnal cortisol rhythmicity and suppressed catecholamine output, reducing basal metabolic rate and promoting hypokinesia [7,32].

The paradox of overweight conferring protection, except in severe oversleeping, involves adipokine-endocrine crosstalk. Adipose tissue in overweight individuals secretes estrogen precursors that undergo aromatization to 17β-estradiol, which in postmenopausal women activates ERα receptors to suppress ubiquitin ligases and enhance Protein kinase B (PKB/Akt) phosphorylation [33,34]. Adiponectin from subcutaneous fat improves insulin sensitivity and activates Adenosine 5‘-monophosphate (AMP)-activated protein kinase (AMPK), preserving muscle glucose uptake during sleep deprivation [35]. Leptin resistance in overweight states paradoxically benefits muscle by sustaining alpha-motor neuron excitability through Janus kinase 2 (JAK2)/STAT3 signaling at neuromuscular junctions [33]. However, severe oversleeping overrides these advantages through extreme IL-6 elevation, which induces Suppressor of cytokine signaling 3 (SOCS3)-mediated leptin resistance and perixisome proliferator-activated receptor alpha (PPARα)-driven fatty acid oxidation that depletes intramyocellular lipids essential for membrane repair [33].

Adults >65 exhibit heightened glucocorticoid receptor sensitivity in muscle, making them vulnerable to sleep deprivation-induced catabolism [36–38]. Overweight and obesity provide protective amino acid reservoirs by enhancing muscle branched-chain keto acid dehydrogenase activity and adipokine-mediated suppression of myostatin [39,40]. In women, adipose-derived estrogen offsets menopause-related anabolic resistance by modulating regulated in development and DNA damage responses 1 (REDD1) expression, but severe oversleeping elevates C-reactive protein to levels that degrade estrogen receptors [41]. Ultimately, these intersecting pathways—HPA axis dysregulation, inflammatory amplification, mitochondrial dysfunction, and sex-specific endocrine adaptations—create a biological milieu where sleep disturbances and body composition dynamically modulate sarcopenia risk through competing anabolic-catabolic equilibria.

This longitudinal cohort study utilizing the CHARLS database benefits from a nationally representative sample and novel investigation into sleep duration’s moderating role in the BMI-sarcopenia relationship. However, key methodological limitations merit careful consideration. Due to the limitation of data of direct body composition, we employed a validated prediction equation incorporating waist circumference, height, age, and sex to estimate ASM, addressing dimensional biases more robustly than weight-based models, but the absence of direct body composition assessment (Dual energy X-ray absorptiometry or Bioelectric impedance analysis) inherently limits diagnostic precision, underdetecting sarcopenic obesity where high adiposity masks low muscle quality, particularly in individuals with severe ectopic fat infiltration unaccounted for by anthropometric proxies. Furthermore, BMI remains an imperfect metric that conflates lean mass and adiposity, complicating interpretation of the observed “overweight paradox” where high BMI may reflect protective muscle preservation rather than adiposity per se. These constraints, intrinsic to large epidemiological datasets, highlight the need for future mechanistic studies integrating gold-standard body composition measurements to disentangle the complex interplay between sleep architecture, adipose-lean tissue partitioning, and sarcopenia pathogenesis. Moreover, future studies should consider the role of circadian misalignment, which may disrupt metabolic and endocrine rhythms and further contribute to muscle wasting. What’s more, the assessment of sleep duration relied on self-report. Although this method is practical and widely used in large-scale epidemiological studies, it is subject to inherent recall bias and systematic measurement error. Future studies should benefit from incorporating objective sleep measures to validate and complement these subjective findings.

## Conclusion

Abnormal sleep durations including sleep deprivation and oversleeping are linked to a heightened risk of sarcopenia among the older adults. Underweight is associated with the increased sarcopenia risk, and overweight is correlated with the decreased risk of sarcopenia, especially in females with abnormal sleep duration except for severe oversleeping, and obesity is associated with the decreased risk of sarcopenia, especially in adults aged over 65 with sleep deprivation.

## Supporting information

S1 File Supplementary Tables. (DOCX)
