# Supplementary material for: The risk assessment of sleep duration on geriatric sarcopenia and its regulatory role in the effect of BMI index on geriatric sarcopenia based on the CHARLS database
Source: PLoS One. 2026 Mar 20;21(3):e0345257. doi: 10.1371/journal.pone.0345257 (PMC13004363; doi:10.1371/journal.pone.0345257)
Supplement: S1 File — (DOCX) [file pone.0345257.s001.docx]

| Supplementary Table 1 Details of missing data in 2011 | | | | | | | | |
| --- | --- | --- | --- | --- | --- | --- | --- | --- |
| Characteristics | Number of missing | Total | Sleep time groups | | | | | Bonferroni’s P |
|  |  |  | Sufficient sleep | Severe sleep deprivation | Mild sleep deprivation | Mild oversleeping | Severe oversleeping |  |
| Number of people | / | 3728 | 962 | 1014 | 1046 | 607 | 99 | / |
| Waist circumference(cm) | 7 | 3721 | 85.27±11.98 | 83.07±12.10 | 84.49±12.79 | 85.46±11.72 | 83.90±11.94 | 0.704 |
| Education level, n (%) | | | | | | | | <0.001 |
| No formal education illiterate | 2 | 2116 | 511 (53.17) | 657 (64.79) | 527 (50.43) | 353 (58.15) | 68 (68.69) | |
| Elementary school |  | 960 | 262 (27.26) | 247 (24.36) | 265 (25.36) | 162 (26.69) | 24 (24.24) | |
| Middle school |  | 455 | 124 (12.90) | 86 (8.48) | 172 (16.46) | 67 (11.04) | 6 (6.06) | |
| College or above |  | 195 | 64 (6.66) | 24 (2.37) | 81 (7.75) | 25 (4.12) | 1 (1.01) | |
| Laboratory data, mean ± SD | | | | | | | | |
| Glucose (mg/dl) | 835 | 2893 | 112.70±36.62 | 109.54±31.78 | 110.72±35.13 | 111.33±33.75 | 105.39±19.10 | 0.052 |
| Creatinine (mg/dl) | 837 | 2891 | 0.81±0.21 | 0.78±0.17 | 0.80±0.21 | 0.81±0.18 | 0.83±0.20 | 0.001 |
| Total Cholesterol (mg/dl) | 834 | 2894 | 195.83±39.50 | 195.57±38.66 | 195.90±39.57 | 197.00±38.02 | 195.27±37.71 | 0.988 |
| Triglycerides (mg/dl) | 834 | 2894 | 132.07±121.53 | 126.02±86.82 | 123.63±81.99 | 129.85±99.00 | 131.30±91.36 | 0.509 |
| Hdl Cholesterol (mg/dl) | 832 | 2896 | 53.47±16.96 | 50.57±14.39 | 52.19±15.39 | 53.40±15.89 | 51.31±16.09 | 0.668 |
| Ldl Cholesterol (mg/dl) | 835 | 2893 | 119.01±34.41 | 118.86±34.68 | 119.49±35.83 | 119.80±36.29 | 115.46±35.97 | 0.957 |

| Supplementary Table 2 Characteristics of participants for cross-sectional analysis | | | | | | | | | |
| --- | --- | --- | --- | --- | --- | --- | --- | --- | --- |
| Characteristics | Total | Sleep time groups | | | | | | Bonferroni’s P | |
|  |  | Sufficient sleep | Severe sleep deprivation | Mild sleep deprivation | Mild oversleeping | Severe oversleeping | |  | |
| Number of people | 3970 | 1095 | 1089 | 1097 | 460 | 229 | | / | |
| Sex, n (%) |  |  |  |  |  |  | | <0.001 | |
| Female | 2080 (52.39) | 545 (49.77) | 659 (60.51) | 540 (49.23) | 216 (46.96) | 120 (52.40) | |  | |
| Male | 1890 (47.61) | 550 (50.23) | 430 (39.49) | 557 (50.77) | 244 (53.04) | 109 (47.60) | |  | |
| Age (year), mean ± SD | 3970 | 63.00 (61.00, 66.00) | 64.00 (62.00, 69.00) | 67.00 (62.00, 71.00) | 69.00 (66.00, 73.00) | 69.00 (65.00, 72.00) | | <0.001 | |
| BMI (Kg/m^2^), mean ± SD | 3970 | 23.28 (20.91, 25.70) | 22.25 (20.08, 24.88) | 22.83 (20.45, 25.42) | 22.81 (20.62, 25.67) | 22.78 (20.56, 24.83) | | <0.001 | |
| Waist circumference(cm), mean ± SD | 3970 | 85.90 (78.20, 93.00) | 83.00 (76.60, 90.00) | 85.20 (78.00, 92.80) | 85.35 (78.00, 93.52) | 86.20 (78.40, 92.40) | | <0.001 | |
| Residence, n (%) <0.001 | | | | | | | | |  |
| rural | 2526 | 641 (66.63) | 715 (70.51) | 661 (63.19) | 428 (70.51) | 81 (81.82) | | | |
| urban | 1202 | 321 (33.37) | 299 (29.49) | 385 (36.81) | 179 (29.49) | 18 (18.18) | | | |
| Education level, n (%) <0.001 | | | | | | | | |  |
| No formal education illiterate | 2118 | 511 (53.17) | 658 (64.89) | 528 (50.48) | 353 (58.15) | 68 (68.69) | | | |
| Elementary school | 960 | 262 (27.26) | 247 (24.36) | 265 (25.36) | 162 (26.69) | 24 (24.24) | | | |
| Middle school | 455 | 124 (12.90) | 86 (8.48) | 172 (16.46) | 67 (11.04) | 6 (6.06) | | | |
| College or above | 195 | 64 (6.66) | 24 (2.37) | 81 (7.75) | 25 (4.12) | 1 (1.01) | | | |
| Marital status, n (%) |  |  |  |  |  |  | <0.001 | | |
| Unmarried | 687 (17.30) | 143 (13.06) | 212 (19.47) | 186 (16.96) | 90 (19.57) | 56 (24.45) | | | |
| Married | 3283 (82.70) | 952 (86.94) | 877 (80.53) | 911 (83.04) | 370 (80.43) | 173 (75.55) | | | |
| Glucose (mg/dl), mean ± SD | 3970 | 103.68 (96.12, 115.96) | 103.14 (95.22, 114.12) | 102.42 (95.76, 113.40) | 103.50 (96.48, 115.56) | 103.50 (94.59, 114.88) | | 0.52 | |
| Creatinine (mg/dl), mean ± SD | 3970 | 0.79 (0.68, 0.92) | 0.76 (0.66, 0.88) | 0.78 (0.67, 0.92) | 0.79 (0.69, 0.93) | 0.78 (0.68, 0.90) | | <0.001 | |
| Total Cholesterol (mg/dl), mean ± SD | 3970 | 192.53 (169.72, 216.50) | 194.07 (170.10, 219.59) | 192.14 (168.27, 218.82) | 191.37 (167.78, 214.56) | 192.33 (173.87, 217.75) | | 0.44 | |
| Triglycerides (mg/dl), mean ± SD | 3970 | 107.08 (73.46, 156.65) | 103.54 (75.22, 147.79) | 101.78 (75.22, 146.69) | 97.35 (74.12, 147.79) | 104.87 (77.00, 151.11) | | 0.37 | |
| Hdl Cholesterol (mg/dl), mean ± SD | 3970 | 48.33 (39.43, 59.92) | 51.80 (41.75, 61.86) | 50.26 (40.98, 60.70) | 50.64 (39.05, 60.79) | 49.10 (39.72, 63.11) | | 0.02 | |
| Ldl Cholesterol (mg/dl), mean ± SD | 3970 | 118.30 (96.17, 138.89) | 117.14 (95.49, 139.56) | 117.53 (95.10, 140.63) | 115.98 (96.07, 137.82) | 117.14 (97.23, 137.73) | | 1.00 | |
| CRP (mg/L) | 3970 | 1.11 (0.59, 2.25) | 1.07 (0.57, 2.17) | 1.07 (0.61, 2.23) | 1.18 (0.62, 2.39) | 1.22 (0.61, 2.32) | |  | |
| Physical activity, n (%) |  |  |  |  |  |  | | 0.629 | |
| No | 725 (18.26) | 207 (18.90) | 173 (15.89) | 214 (19.51) | 87 (18.91) | 44 (19.21) | |  | |
| Light | 1028 (25.89) | 281 (25.66) | 275 (25.25) | 285 (25.98) | 128 (27.83) | 59 (25.76) | |  | |
| Moderate | 1113 (28.04) | 307 (28.04) | 314 (28.83) | 299 (27.26) | 124 (26.96) | 69 (30.13) | |  | |
| Vigorous | 1104 (27.81) | 300 (27.40) | 327 (30.03) | 299 (27.26) | 121 (26.30) | 57 (24.89) | |  | |

| Supplementary Table 3 Cox regression results of the relationship between sleep duration and sarcopenia stratified by sex | | | | | | | | | | | | | |  |
| --- | --- | --- | --- | --- | --- | --- | --- | --- | --- | --- | --- | --- | --- | --- |
| Characteristics | Model 1 | | | Model 2 | | | Model 3 | | | P for interaction | | | |  |
|  | HR | 95% CI | P | HR | 95% CI | P | HR | 95% CI | P | |  | | | |
| Sex |  | | |  | | |  |  |  | | 0.168 | | | |
| Female |  | | |  | | |  |  |  | |  | | | |
| Sufficient sleep | ref | | | ref | | | ref | | |  | | | |  |
| Severe sleep deprivation | 1.78 | 1.25, 2.54 | 0.001 | 1.40 | 0.98, 2.01 | 0.065 | 1.44 | 1.01, 2.06 | 0.048 | | | |  |  |
| Mild sleep deprivation | 1.54 | 1.05, 2.25 | 0.028 | 1.49 | 1.02, 2.18 | 0.041 | 1.49 | 1.02, 2.19 | 0.041 | | | |  |  |
| Mild oversleeping | 1.57 | 0.97, 2.53 | 0.065 | 1.55 | 0.95, 2.50 | 0.078 | 1.60 | 0.988, 2.60 | 0.056 | | | |  |  |
| Severe oversleeping | 2.76 | 1.71, 4.45 | <0.001 | 2.30 | 1.42, 3.74 | <0.001 | 2.06 | 1.27, 3.36 | 0.004 | | | |  |  |
| Male | | | | | | |  |  |  | |  | | | |
| Sufficient sleep | ref | | | ref | | | ref | | |  | | | |  |
| Severe sleep deprivation | 1.33 | 1.02, 1.74 | 0.034 | 1.13 | 0.86, 1.48 | 0.381 | 1.10 | 0.84, 1.44 | 0.499 | | |  | |  |
| Mild sleep deprivation | 1.24 | 0.96, 1.60 | 0.098 | 1.22 | 0.95, 1.58 | 0.125 | 1.21 | 0.94, 1.56 | 0.150 | | |  | |  |
| Mild oversleeping | 1.52 | 1.12, 2.05 | 0.006 | 1.39 | 1.02, 1.88 | 0.035 | 1.39 | 1.02, 1.88 | 0.040 | | | |  |  |
| Severe oversleeping | 1.31 | 0.87, 1.98 | 0.195 | 1.13 | 0.75, 1.71 | 0.566 | 1.13 | 0.74, 1.70 | 0.573 | | | |  |  |
| HR, Hazard Ratio; CI, Confidence Interval.  Model 1 was not adjusted;  Model 2 was adjusted for age, sex, marital status, education level, residence status, BMI, waist circumstance and physical activity;  Model 3 was further adjusted for glucose, creatinine, HDL-c, LDL-c, TG, and CRP based on Model 2. | | | | | | | | | | | | | |  |

| Supplementary Table 4 Association between BMI and risk for sarcopenia, grouped by sex, stratified by sleep duration | | | | | | | | | | | | |
| --- | --- | --- | --- | --- | --- | --- | --- | --- | --- | --- | --- | --- |
| Characteristics | Female | | |  | | Male | | |  |  | No. of cases/total (female) | No. of cases/total (male) |
|  | HR | 95%CI | P |  |  | HR | 95%CI | P |  |  |  |  |
| All participants | | | | |  | | | |  |  |  |  |
| Normal BMI | ref | | |  | | ref | | |  |  | 181/940 | 287/1082 |
| Underweight | 1.70 | 1.23, 2.35 | 0.001 |  | | 1.73 | 1.31, 2.29 | <0.001 |  |  | 66/180 | 66/159 |
| Overweight | 0.11 | 0.06, 0.20 | <0.001 |  | | 0.91 | 0.69, 1.20 | 0.514 |  |  | 14/596 | 81/418 |
| Obesity | 0.00 | 0.00, Inf | 0.989 |  | | 0.87 | 0.53 1.44 | 0.588 |  |  | 0/234 | 19/119 |
| Sufficient sleep | | | | |  | | | |  |  |  |  |
| Normal BMI | ref | | |  | | ref | | |  |  | 27/222 | 58/276 |
| Underweight | 1.91 | 0.84, 4.34 | 0.120 |  | | 2.13 | 1.07, 4.25 | 0.031 |  |  | 10/34 | 13/31 |
| Overweight | 0.09 | 0.02, 0.42 | 0.002 |  | | 0.74 | 0.41, 1.32 | 0.302 |  |  | 3/162 | 24/130 |
| Obesity | 0.00 | 0.00, Inf | 0.997 |  | | 0.42 | 0.15, 1.21 | 0.107 |  |  | 0/65 | 6/42 |
| Severe deprivation | | | | |  | | | |  |  |  |  |
| Normal BMI | ref | | |  | | ref | | |  |  | 63/315 | 72/262 |
| Underweight | 1.74 | 1.00, 3.03 | 0.049 |  | | 1.43 | 0.82, 2.51 | 0.208 |  |  | 26/70 | 17/41 |
| Overweight | 0.08 | 0.03, 0.26 | <0.001 |  | | 0.91 | 0.51, 1.65 | 0.765 |  |  | 4/170 | 19/75 |
| Obesity | 0.00 | 0.00, Inf | 0.994 |  | | 0.39 | 0.09, 1.66 | 0.200 |  |  | 0/62 | 1/19 |
| Mild sleep deprivation | | | | |  | | | |  |  |  |  |
| Normal BMI | ref | | |  | | ref | | |  |  | 30/243 | 88/324 |
| Underweight | 2.08 | 1.01, 4.29 | 0.048 |  | | 1.62 | 0.95, 2.75 | 0.076 |  |  | 15/45 | 20/52 |
| Overweight | 0.15 | 0.06, 0.40 | <0.001 |  | | 0.97 | 0.57, 1.63 | 0.895 |  |  | 5/158 | 23/123 |
| Obesity | 0.00 | 0.00, Inf | 0.996 |  | | 0.70 | 0.26, 1.88 | 0.484 |  |  | 0/66 | 6/35 |
| Mild oversleeping | | | | |  | | | |  |  |  |  |
| Normal BMI | ref | | |  | | ref | | |  |  | 30/133 | 58/191 |
| Underweight | 2.96 | 1.01, 8.69 | 0.049 |  | | 1.69 | 0.79, 3.60 | 0.176 |  |  | 12/25 | 14/28 |
| Overweight | 0.00 | 0.00, Inf | 0.998 |  | | 0.56 | 0.25, 1.25 | 0.156 |  |  | 1/93 | 14/80 |
| Obesity | 0.00 | 0.00, Inf | 0.999 |  | | 0.98 | 0.25, 3.84 | 0.974 |  |  | 0/37 | 3/20 |
| Severe oversleeping | | | | |  | | | |  |  |  |  |
| Normal BMI | ref | | |  | | ref | | |  |  | 11/27 | 11/29 |
| Underweight | 1.87 | 0.59, 5.96 | 0.291 |  | | 2.34 | 0.70, 7.77 | 0.166 |  |  | 3/6 | 2/7 |
| Overweight | 0.35 | 0.06, 1.91 | 0.225 |  | | 0.62 | 0.16, 2.37 | 0.485 |  |  | 1/13 | 1/10 |
| Obesity | 0.00 | 0.00, Inf | 0.999 |  | | 3.96 | 0.75, 20.95 | 0.106 |  |  | 0/4 | 3/3 |
| HR, Hazard Ratio; CI, Confidence Interval.  Models were adjusted for age, sex, marital status, education level, residence status, waist circumstance, physical activity, glucose, creatinine, HDL-c, LDL-c, TG, and CRP. | | | | | | | | | | | | |

| Supplementary Table 5 Association between BMI and risk for sarcopenia, grouped by age median, stratified by sleep duration | | | | | | | | |
| --- | --- | --- | --- | --- | --- | --- | --- | --- |
| Characteristics | 60-65 | | | >65 | | | No. of cases/total (60-65) | No. of cases/total (>65) |
|  | HR | 95%CI | P | HR | 95%CI | P |  |  |
| All participants | | | | | | |  |  |
| Normal BMI | ref | | | ref | | | 151/1034 | 317/988 |
| Underweight | 2.25 | 1.46, 3.47 | <0.001 | 1.72 | 1.36, 2.18 | <0.001 | 40/136 | 92/203 |
| Overweight and obesity | 0.57 | 0.37, 0.88 | 0.010 | 0.43 | 0.32, 0.58 | <0.001 | 40/580 | 55/434 |
| Obesity | 0.25 | 0.09, 0.64 | 0.004 | 0.30 | 0.17, 0.53 | <0.001 | 7/200 | 12/153 |
| Sufficient sleep | | | | | | |  |  |
| Normal BMI | ref | | | ref | | | 47/367 | 38/131 |
| Underweight | 2.32 | 1.06, 5.11 | 0.036 | 2.82 | 1.45, 5.48 | 0.002 | 10/40 | 13/25 |
| Overweight | 0.08 | 0.02, 0.39 | 0.002 | 0.40 | 0.19, 0.87 | 0.021 | 19/227 | 8/65 |
| Obesity | 0.00 | 0.00, Inf | 0.996 | 0.27 | 0.07, 1.00 | 0.050 | 4/81 | 2/26 |
| Severe deprivation | | | | | | |  |  |
| Normal BMI | ref | | | ref | | | 55/349 | 80/228 |
| Underweight | 2.17 | 1.28, 3.69 | 0.004 | 1.25 | 0.82, 1.90 | 0.307 | 15/44 | 28/67 |
| Overweight | 0.08 | 0.02, 0.25 | <0.001 | 0.30 | 0.16, 0.59 | <0.001 | 13/165 | 10/80 |
| Obesity | 0.00 | 0.00, Inf | 0.993 | 0.08 | 0.01, 0.59 | 0.013 | 1/58 | 0/23 |
| Mild sleep deprivation | | | | | | |  |  |
| Normal BMI | ref | | | ref | | | 32/227 | 106/340 |
| Underweight | 2.19 | 1.08, 4.41 | 0.029 | 1.68 | 1.03, 2.73 | 0.036 | 11/36 | 24/61 |
| Overweight | 0.14 | 0.05, 0.38 | <0.001 | 0.63 | 0.38, 1.05 | 0.074 | 7/140 | 21/141 |
| Obesity | 0.00 | 0.00, Inf | 0.996 | 0.24 | 0.09, 0.70 | 0.009 | 2/41 | 4/60 |
| Mild oversleeping | | | | | | |  |  |
| Normal BMI | ref | | | ref | | | 15/72 | 73/242 |
| Underweight | 4.51 | 1.67, 12.19 | 0.003 | 2.76 | 1.48, 5.12 | 0.001 | 4/13 | 22/40 |
| Overweight | 0.00 | 0.00, Inf | 0.998 | 0.30 | 0.14, 0.63 | 0.001 | 1/45 | 14/128 |
| Obesity | 0.00 | 0.00, Inf | 0.999 | 0.38 | 0.10, 1.40 | 0.145 | 0/19 | 3/38 |
| Severe oversleeping | | | | | | |  |  |
| Normal BMI | ref | | | ref | | | 2/9 | 20/47 |
| Underweight | 1.84 | 0.59, 5.81 | 0.296 | 2.14 | 0.96, 4.78 | 0.064 | 0/3 | 5/10 |
| Overweight | 0.34 | 0.06, 1.76 | 0.198 | 0.64 | 0.24, 1.69 | 0.364 | 0/3 | 2/20 |
| Obesity | 0.00 | 0.00, Inf | 0.998 | 2.21 | 0.57, 8.54 | 0.249 | 0/1 | 3/6 |
| HR, Hazard Ratio; CI, Confidence Interval.  Models were adjusted for age, sex, marital status, education level, residence status, waist circumstance, physical activity, glucose, creatinine, HDL-c, LDL-c, TG, and CRP. | | | | | | | | |
